# Supplementary figures and images for: Self-limiting stem-cell niche signaling through degradation of a stem-cell receptor
Source: PLoS Biol. 2020 Dec 14;18(12):e3001003. doi: 10.1371/journal.pbio.3001003 (PMC7769618; doi:10.1371/journal.pbio.3001003)

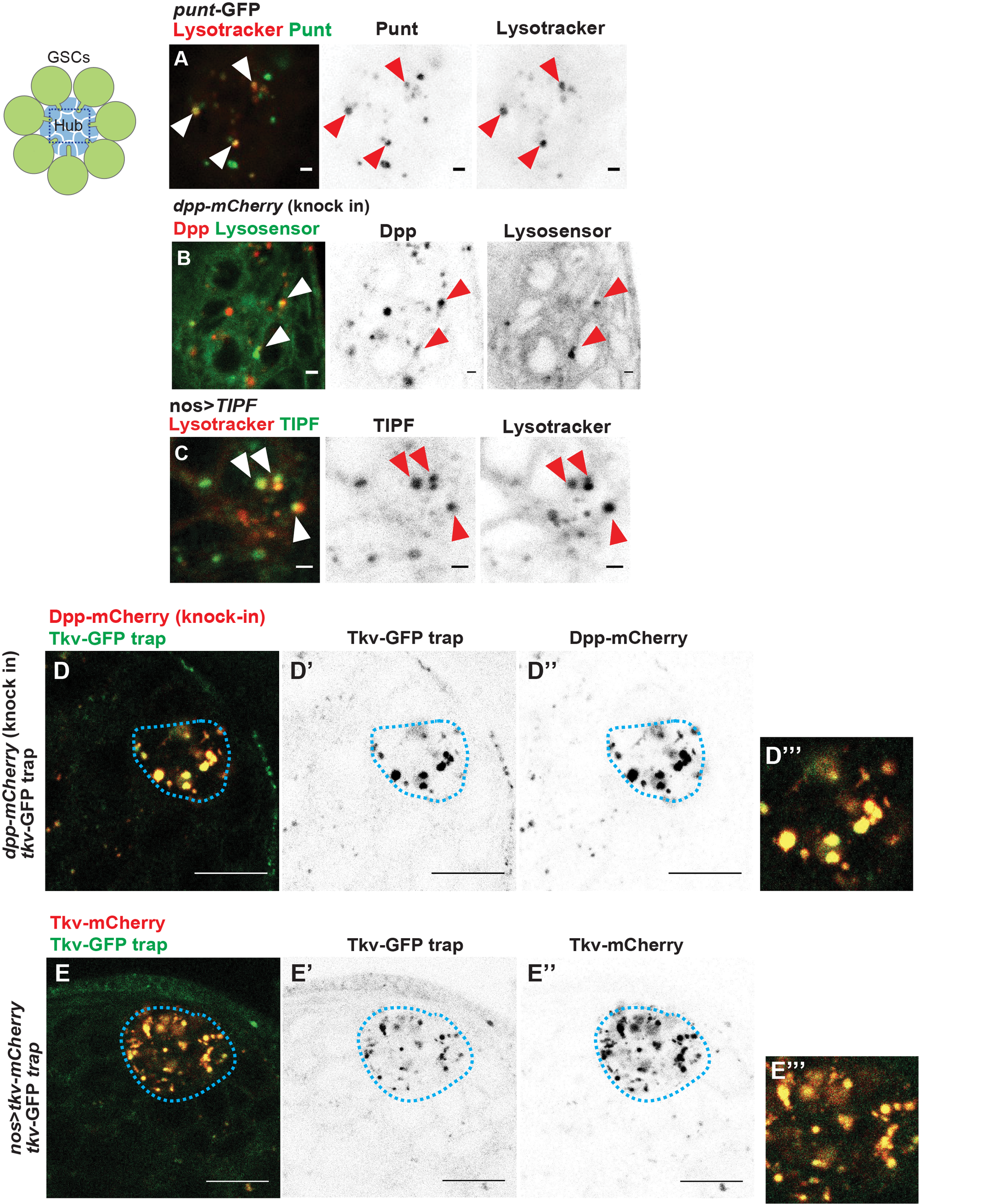

Supplement: S1 Fig — A, B, C, Representative images of the hub area of indicated genotypes. Arrowheads indicate the colocalization of Punt (A), Dpp (B), and TIPF (C) with the hub lysosomes (marked by lysotracker in A and C, lysosensor in B). D, E, Representative images of the testis tip of indicated genotypes. D, The Dpp-mCherry (CRISPR knock-in) signal colocalizes with the Tkv-GFPtrap signal in the hub. E, Tkv-mCherry expressed in the germline colocalizes with the Tkv-GFPtrap signal in the hub. D”‘ and E”‘ show magnified hub areas. Scale bars; 1 μm in A, B, C and 10 μm in other images. Blue dotted lines outline the hub. All experiments in this Fig were performed using live tissues. Dpp, Decapentaplegic. (TIF) [file pbio.3001003.s001.tif]

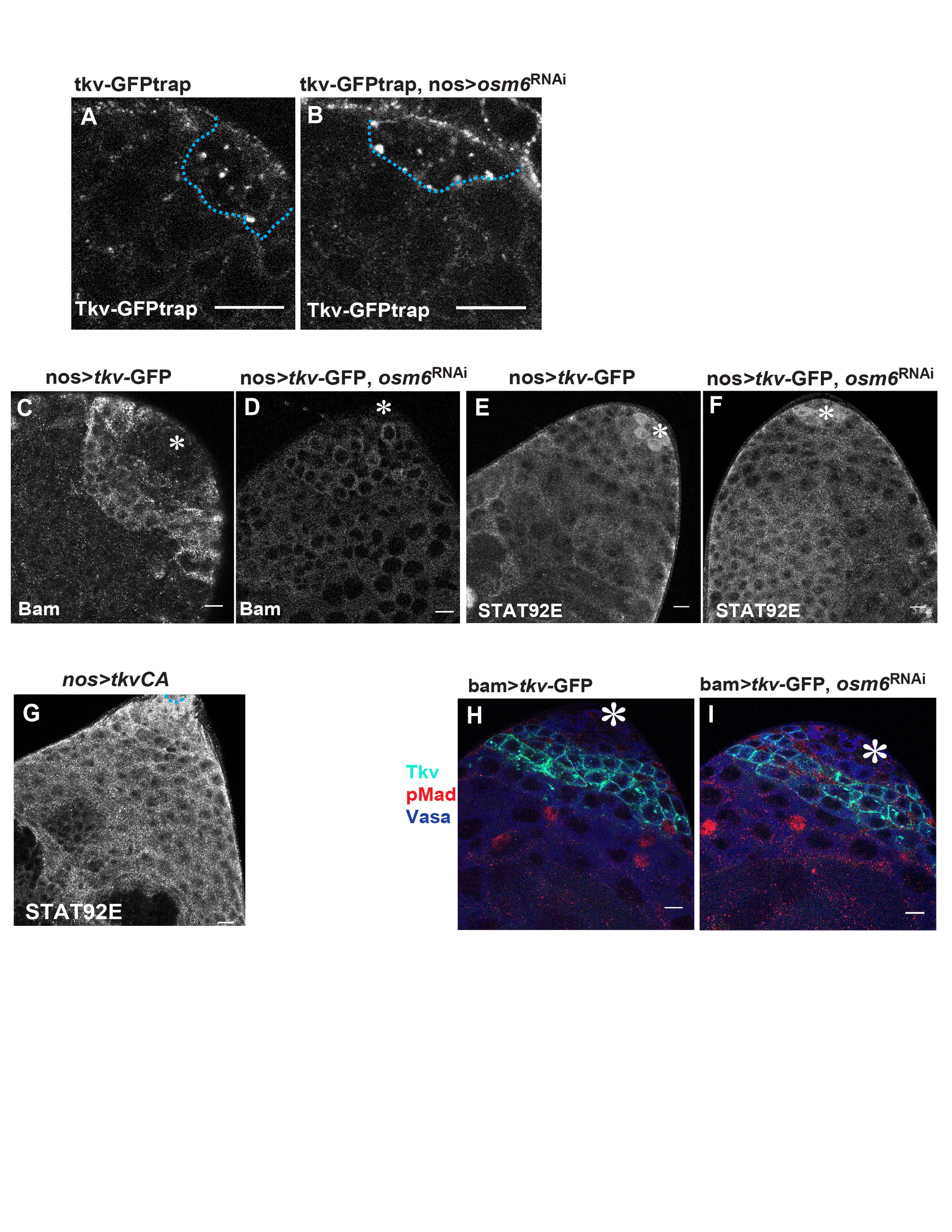

Supplement: S2 Fig — A, B, Representative images of testes tips of Tkv-GFPtrap flies without (A) or with (B) IFT-KD (nosGal4>osm6 RNAi). Blue dotted lines outline the hub. C–F, Representative Bam staining (C, D) and STAT92E staining (E, F) of testes expressing Tkv-GFP with (C, E, nosGal4>tkv-GFP) or without (D, F, nosGal4>tkv-GFP, osm6 RNAi) IFT (osm6) RNAi. G, Representative image of STAT92E staining of the testis expressing a constitutive active form of Tkv (nosGal4>tkv-CA). H, I, Representative images of bamGal4-mediated expression of Tkv-GFP with (H, bamGal4>tkv-GFP) or without (I, bamGal4>tkv-GFP, osm6 RNAi) IFT (osm6) RNAi. Vasa (blue), pMad (red), Tkv (green). Scale bars, 10 μm. Asterisks indicate the approximate location of the hub. For A and B, imaging was performed using live tissues. Fixed samples were used for C–I. IFT, intraflagellar transport; pMad, phosphorylated Mad; RNAi, RNA interference; Tkv, Thickveins. (TIF) [file pbio.3001003.s002.tif]

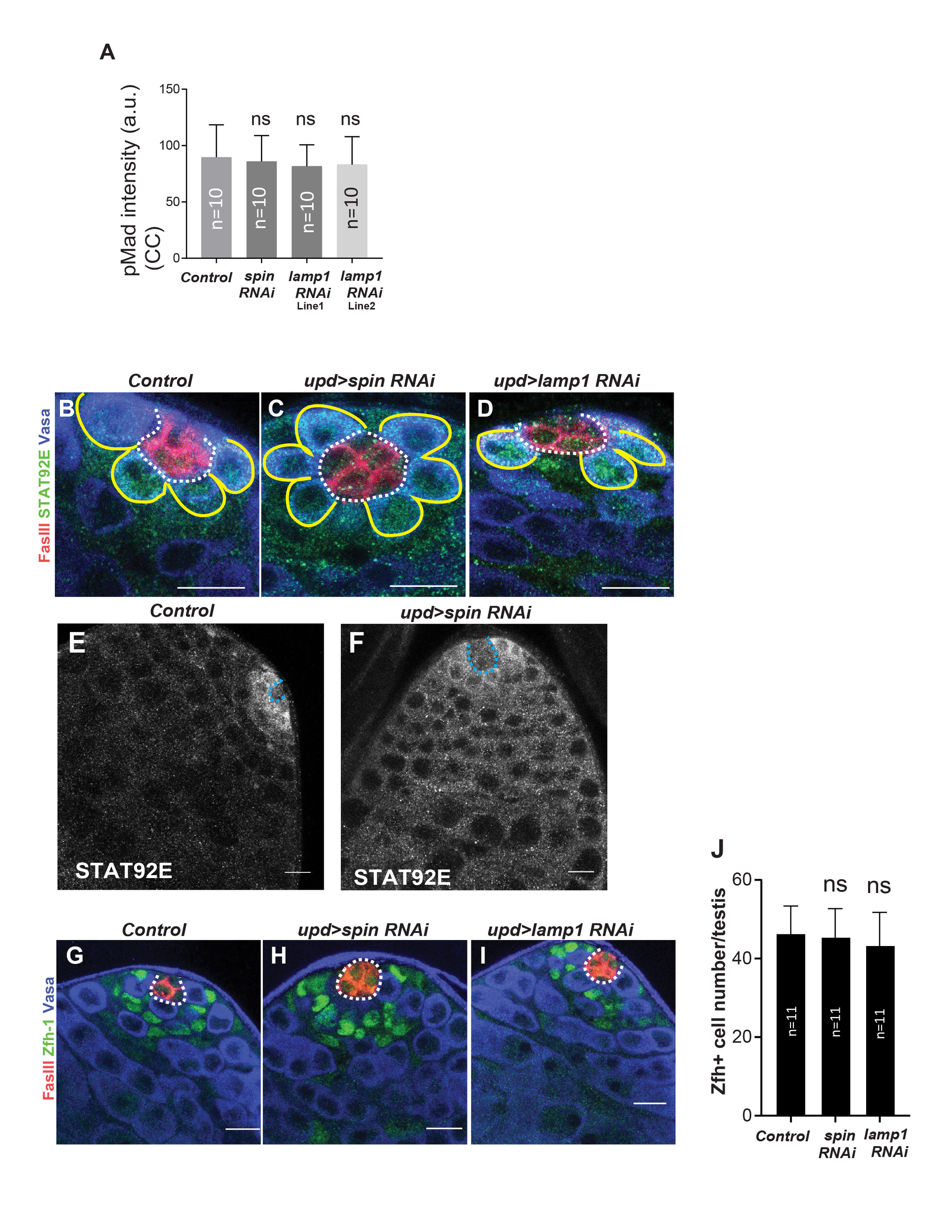

Supplement: S3 Fig — A, Quantification of pMad intensity in the somatic CCs (yellow arrows in Fig 3 A–D). Average intensity of CCs in the entire testis was scored from 10 testes for each genotype. ns; nonsignificant (p≥0.05), from Dunnett multiple comparisons test. B–D, Representative images of STAT92E (green) staining in the testis of indicated genotypes. FasIII (red, hub marker), Vasa (blue, germline marker). White dotted lines outline the hub. Yellow lines outline GSCs. E, F, Representative images of STAT92E staining of a broader area of the testis from indicated genotypes. Blue dotted lines outline the hub. G–I, Representative images of the testis tip of indicated genotypes. Zfh-1 (green, CySC marker), FasIII (red, hub marker), Vasa (blue, germline marker). J, Number of Zfh-1-positive CySCs in the indicated genotypes. Testes (n = 11) from 2 independent experiments were scored for each group. ns; nonsignificant (p≥0.05), from Dunnett multiple comparisons test. Fixed samples were used for all experiments. Underlying numerical data for A and J are provided in S1 Data. CC, cyst cell; CySC, somatic cyst stem cell; GSC, germline stem cell; pMad, phosphorylated Mad. (TIF) [file pbio.3001003.s003.tif]

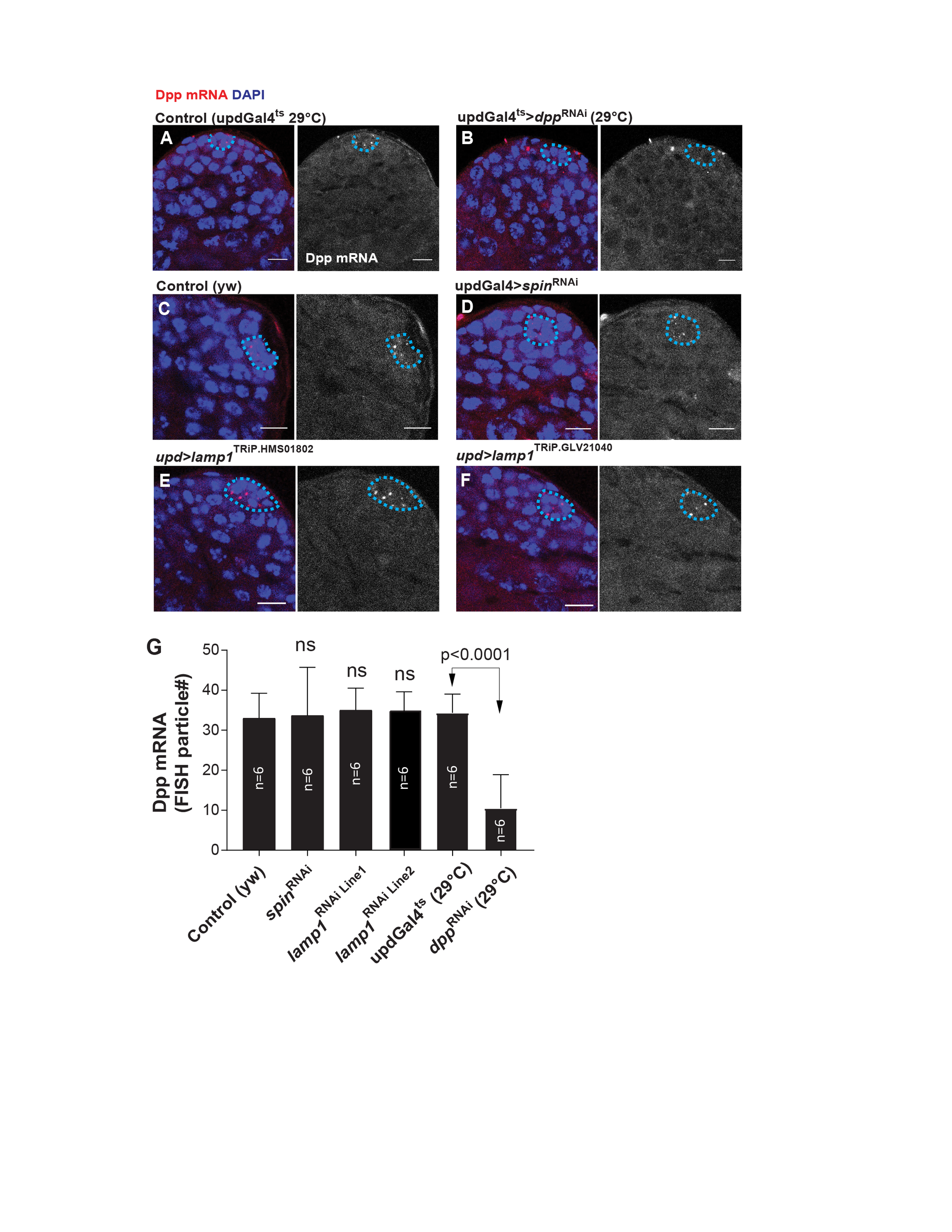

Supplement: S4 Fig — A–G, Representative images of in situ hybridization using a Stellaris FISH probe against dpp mRNA (red) in the testes of indicated genotypes. B, The Dpp RNAi (negative control) testis shows almost no detectable signal in the hub, indicating the specificity of the probe. The hub is encircled by a blue dotted line. DAPI (blue) marks nuclei. Scale bars, 10 μm. G, Number of particles of Dpp FISH in the hub of indicated genotypes (see Methods). Data are means and standard deviations. Testes (n = 6) from 2 independent experiments were scored for each group. The adjusted P value from Dunnett multiple comparisons test is provided. ns; nonsignificant (p≥0.05). Underlying numerical data for G are provided in S1 Data. Dpp, Decapentaplegic; FISH, fluorescence in situ hybridization; RNAi, RNA interference. (TIF) [file pbio.3001003.s004.tif]

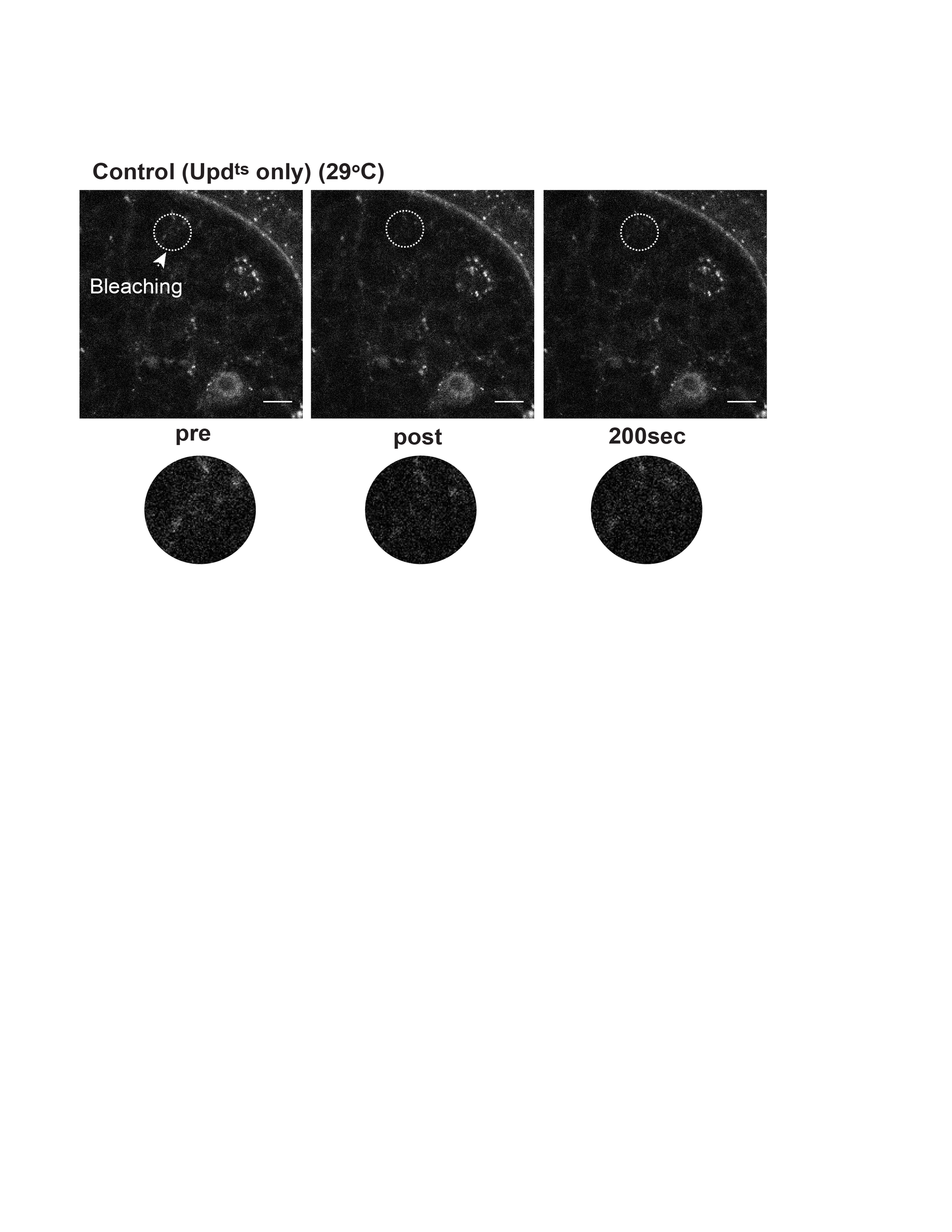

Supplement: S5 Fig — A representative FRAP experiment of the testis tip of a control testis (updGal4ts line, after a 4-day temperature shift) in which Dpp-mCherry is not expressed. A region encircled by a white dotted line was photobleached, and the intensity of the mCherry signal was monitored before and after photobleaching at the indicated time points. Lower panels are magnifications of the white dotted circles. Scale bars; 10 μm. Live tissues were used for imaging. Dpp, Decapentaplegic; FRAP, fluorescence recovery after photobleaching. (TIF) [file pbio.3001003.s005.tif]

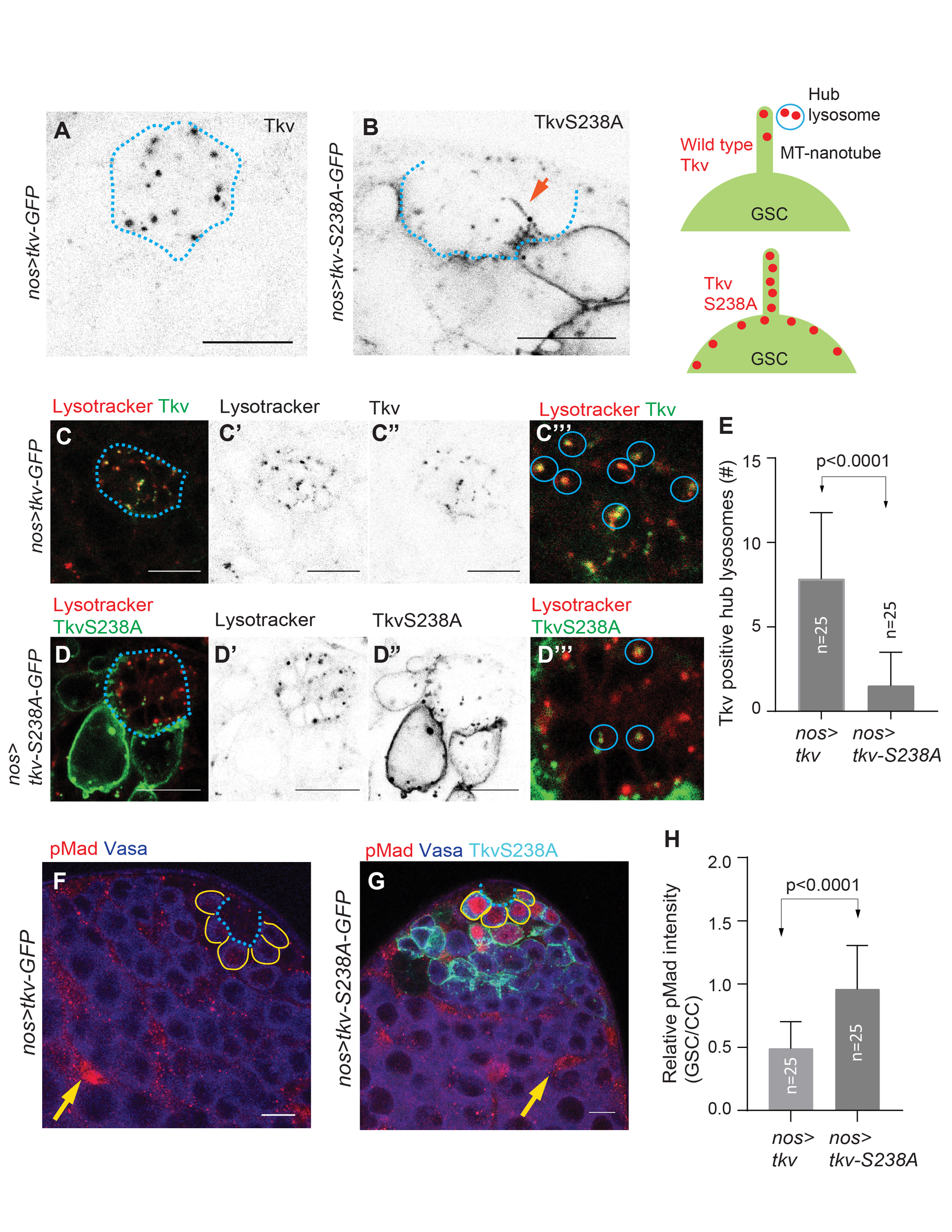

Supplement: S6 Fig — A, B, Representative images of testis tips with nosGal4-mediated expression of tkv-GFP (A) or tkvS238A-GFP (B). Blue dotted lines outline the hub. The arrow in B indicates a MT-nanotube decorated with TkvS238A. The right panel explains the difference between Tkv and Tkv-S238A’s localization pattern. C, D, Representative images of testis tips with nosGal4-mediated expression of tkv-GFP (C) or tkvS238A-GFP (D) (green) with lysotracker staining (red). Blue dotted lines outline the hub. C”‘ and D”‘ show magnified images of the hub area. Lysotracker-positive lysosomes (>0.5 μm diameter) positive with Tkv are marked by blue circles. E, Number of Tkv-positive hub lysosomes in the indicated genotypes. Lysosomes in the entire hub region were counted as lysotracker-positive punctae >0.5 μm diameter from z-stacks collected at 0.5 μm intervals. Total testes (n = 25) from 2 independent experiments were scored for each group. F, G, Representative images of pMad staining in testes with nosGal4-mediated expression of Tkv-GFP (F) or TkvS238A-GFP (G). Blue dotted lines outline the hub. Yellow lines outline GSCs. Vasa (blue), pMad (red), Tkv (green, note that Tkv overlapping with Vasa appears as cyan). Arrows indicate CCs used as an internal control (see Methods). H, Quantification of pMad intensity in GSCs (relative to CCs, yellow arrows in F and G) of nosGal4-mediated Tkv- or TkvS238A-expressing testes. GSCs (n = 25) from 2 independent experiments were scored for each group. Scale bars are 10 μm in all images. For E and H, P values were calculated by Student t tests. For A–E, imaging and measurements were performed using live tissues. Fixed samples were used for F–H. Underlying numerical data for E and H are provided in S1 Data. CC, cyst cell; GSC, germline stem cell; MT-nanotube, microtubule-based nanotube; pMad, phosphorylated Mad; Tkv, Thickveins. (TIF) [file pbio.3001003.s006.tif]
