# Supplementary material for: Self-limiting stem-cell niche signaling through degradation of a stem-cell receptor
Source: PLoS Biol. 2020 Dec 14;18(12):e3001003. doi: 10.1371/journal.pbio.3001003 (PMC7769618; doi:10.1371/journal.pbio.3001003)
Supplement: Attachment — Submitted filename: Response.docx [file pbio.3001003.s012.docx]

**Below, reviewer comments are in black. Editor comments, annotation and advice are in red. Our responses are in blue.**

**Editor comments:**
Thank you very much for submitting your manuscript "Stem-cell niche self-restricts the signaling range via receptor-ligand degradation" for consideration as a Short Reports at PLOS Biology. Your manuscript has been evaluated by the PLOS Biology editors, an Academic Editor with relevant expertise, and by four independent reviewers.

In light of the reviews (below), we will not be able to accept the current version of the manuscript, but we would welcome re-submission of a revised version that takes into account the reviewers' comments. Our Academic Editor and we have categorised the concerns into four groups and provided guidance on how to address them. You will find this in an attachment to this mail. In principle, we are happy for textual revisions for the concerns that can be accomplished in that way, but there are several points that the reviewers and editors feel need additional data to be convincing. We cannot make any decision about publication until we have seen the revised manuscript and your response to the reviewers' comments. Your revised manuscript is also likely to be sent for further evaluation by the reviewers.

**Annotated comments with advice on revisions:** *Our Academic Editor and we have categorized the concerns into four groups and provided guidance on how to address them. Immediately below are the four categories. Further below are the comments from the reviewers, which are annotated using the categorization with additional advice in some places. We have bolded the parts of reviewers’ comments to indicate which text is annotated.*

A. *Quantitation of the amount of Dpp or Tkv in particular cells or conditions, including of their degradation as you proposed. You will need to address this, which likely requires new data.*
B. *Cellular precision as to which cell types, or where in respect to the cells, the Dpp or Tkv is going. You will need to address this, which likely requires new data.*
C. *The concern about the lysosomal part of the paper, which would be ideal to address.*
D. *At least two reviewers raised a concern about consistency with your prior reports. You will need to address it, which could be with text modifications.*

We would like to thank you and the academic editor for taking the time to extract all fundamental issues as well as categorizing and annotating all reviewers’ concerns/suggestions. Your advice tremendously helped us improve the manuscript. We also would like to thank all the reviewers for reading our manuscript carefully and providing us with critical comments and suggestions. This revision process has led even us ourselves understand our data at a deeper level.

Here, we first would like to summarize the major concerns raised by multiple reviewers. These issues are partly because of our writing, and partly because of the lack of data. First, we will clarify these major issues here, such that point-by-point responses below will be easier to follow.

**#1. Reviewers raised the concern that increased Tkv in the lysosome upon inactivation of lysosomes might not contribute to Tkv signaling because it is already in lysosomes (Annotated as A or C)**

This is indeed an important point, and raised by reviewer 1, 2, and 4. In the original manuscript we only showed that inhibition of the hub lysosomes led to an increased amount of Tkv in the hub (without precisely distinguishing in what compartment Tkv may be in), and we inferred that this may mean increased Tkv-mediated signaling. However, the reviewers are right to question how Tkv can participate in signaling if they are in the lysosomes.

This comment made us realize the meaning of an observation we had noted for quite some time. The data is now incorporated as a new Figure 2 (below), as we believe this addresses this major issue raised by multiple reviewers. We had noted Tkv-GFPtrap shows a thread-like pattern in the hub, which increases in frequency upon CQ treatment (Figure S1A). This is likely a Tkv population that is still present on MT-nanotubes (i.e. signal-competent), as co-labelling of Tkv and lysosomes shows that thread-like Tkv is typically NOT associated with lysosomes (Figure S1A).

This time, we scored the frequency of Tkv threads and found that the percentage of the hub containing any Tkv-thread was significantly increased upon inactivation of hub lysosomes. These data suggest that inactivation of hub lysosomes increases Tkv that is still on MT-nanotubes (in the hub area), but not associated with lysosomes.

Previously, we did not think of the way to distinguish two Tkv populations; in the hub and associated with lysosomes vs. in the hub but not associated with lysosomes. Now reviewers’ question made us realize the importance of distinguishing these distinct Tkv populations, and we hope that this added figure addresses their concern.

(Note that we have not succeed in visualizing the MT-nanotubes together with hub specific lysosomal inhibition due to technical difficulties. Further studies will be necessary such as generation of new genetic tools and/or improving our imaging method).

**F-H,** Examples of the thread pattern of the tkv-GFPtrap signal (Tkv threads) seen in the hub of indicated genotypes. Blue dotted lines outline the hub. Arrowheads indicate Tkv threads seen after the knockdown of hub lysosomal genes (*spin, lamp1*). Magnified portions of the hub are shown in right-hand panels. **I,** Representative images of a Tkv thread (encircled by a green dotted line in **I’**) in a portion of the hub of a *spin* RNAi fly. Tkv threads are typically lysotracker negative. Arrowheads indicate the starting and ending points of the thread. Tkv-GFPtrap (green), Lysotracker (red). **J,** Frequency of hubs containing any Tkv threads in each genotype. Indicated numbers of testes from two independent experiments were scored for each group. Entire hub areas were imaged for scoring using 0.5 μm interval z-stacks. Image in **I** is a portion of the hub marked as a blue box in the left-hand panel**.**

Blue dotted lines outline the hub. Lower panels are magnified images of the threads. Arrowheads indicate ending points of the threads. Tkv-GFPtrap (green), Lysotreacker (red). Scale bars; 1μm in **I**, 10μm in other images.

**#2. Reviewers raised alternative possibilities as to how the inactivation of lysosomes can indirectly increase Dpp/Tkv signaling (Annotated as B or C)**

This concern was linked partly to the above point #1 that the Tkv in the lysosome would not participate in signaling. Also, thus, the observed increase in pMad may be caused indirectly by other mechanisms because the interference of lysosome would have a broad effect. Based on these considerations, the reviewers suggested us to examine other aspects of GSCs, which might in turn lead to increased pMad, such as **elevated JAK-STAT signaling and/or the contribution of somatic cyst stem cells (CySCs).**

First, we hope that our response above (#1) partially addresses this concern, as the ‘active Tkv’ pool likely increases upon inhibition of lysosomes in the hub. Second, our data using a Tkv mutant that cannot be ubiquitinated (which we have shown interferes with Tkv transfer to hub cells) also exhibits increased signal activation in GSCs, supporting that Tkv’s degradation is indeed important. However, we also agree with reviewers that other possibilities must be more thoroughly considered, as lysosome inactivation in the hub is expected to have broad impacts.

i) We tested JAK-STAT activation by STAT92E staining in GSCs upon inhibition of hub lysosomes (suggested by reviewers #2 and #4). The nuclear STAT92E staining in GSCs did not show a detectable change after lysosome inactivation (in new Figure S2C-E).

ii) We tested whether the number of CySCs is increased and whether they express higher amounts of Dpp (mRNA) upon lysosome inactivation, either of which could increase pMad levels in GSCs (suggested by reviewer #2). We found that CySC number remained the same in the hub specific knockdown of lysosomal genes. Moreover, we did not detect any Dpp mRNA in CySC populations either in wild-type or knockdown testis (Figure S2H-K, Figure S4).

iii) In addition, we also found that GSC clones with Tkv-OE/IFT-KD cell autonomously increase their pMad levels (new Figure 3K), suggesting that Tkv’s effect is cell autonomous.

Based on these experiments, we propose that the increased Tkv signaling upon hub-lysosome inactivation is mostly due to increased Tkv (as a result of decreased Tkv degradation). However, we also edited the entire text to interpret the results cautiously not to entirely exclude indirect effects, as suggested by reviewers.

**#3. Reviewer #1 and #4 commented that “this manuscript contradicts our previous paper (Inaba et al., Nature 2015).”**

We fully understand that the previous and current paper may appear to contradict each other, and we did not explain the difference very well, leading to the most critical misunderstanding. The apparent contradiction, together with the results leading to each conclusion, are summarized below:

**Previous paper:** The MT-nanotube is required to upregulate Dpp signaling. This conclusion was based on, where MT-nanotube removal (by IFT knockdown) **decreased** the downstream signal activation (pMad level).

**Current paper:** The MT-nanotube is required to downregulate Dpp signaling. IFT knockdown **increased** pMad level **when combined with Tkv overexpression**, leading to tumor formation outside the niche.

The only difference between these conditions that led to an opposite outcome was the presence or absence of Tkv overexpression. To understand how this happens, we consulted our long-standing collaborator Dr. Boris M. Slepchenko, with mathematical modeling expertise. Although we did not have precise parameters to initiate actual modeling, two simple facts, 1) the MT-nanotube surface is 1/150 of the entire plasma membrane (this is a decently accurate estimation based on our confocal imaging, 2) Tkv must meet with the ligand Dpp to signal, are likely sufficient to explain why we obtained opposite outcomes depending on the different Tkv expression level. This is summarized in the provided cartoon figure below (Figure 3L, the following i-iv should correspond to Figure’s i-iv).

i) Under normal conditions, Tkv is trafficked to the surface of MT-nanotubes, where it interacts with Dpp, leading to a ‘normal’ pMad level in the GSC nucleus (Figure 3L-i).

ii) When MT-nanotube formation is compromised, Tkv disperses to the entire cell cortex, which might reduce its effective interaction with Dpp leading to reduced pMad levels. If the same number of molecules A and B are located on the surface of different sizes, the binding probability of A and B is proportional to ~ 1/surface area. Based on our confocal images, the nanotube comprises about 1/150 of the surface of entire cell. If the Tkv destined for the MT-nanotube surface redistributes to the entire cell plasma membrane, we would predict a 1/150 decrease in binding rate of Tkv and Dpp. This also suggests the possibility that MT-nanotubes may contribute to a higher probability of ligand-receptor interaction by concentrating molecules on their surface.

iii) When Tkv is overexpressed, excess Tkv is trafficked to MT-nanotubes and degraded, and no increased pMad is observed (Figure 3L-iii).

iv) When Tkv is overexpressed and the MT-nanotube is compromised, excess Tkv localizes to the entire cell cortex and is no longer degraded. Tkv on the cell cortex now interacts with more of the available Dpp leading to an increased pMad level (Figure 3L-iv).

We have added these explanations to the revised manuscript to clearly explain why our two papers are not contradictory to each other, but complementarily explain the mode of Dpp/Tkv activation.

_________

reviewer 1

First, this appears to be a revised manuscript. If so, I have not seen the previous version, the previous reviews or the authors' response to those reviews. So my apologies if my review covers points that the authors have already discussed.

The manuscript presents novel data that, in the Drosophila testes, the BMP receptor Tkv can be transferred from Germline Stem Cells (GSCs) to the adjacent hub cells via an unknown mechanism that may involve MT-nanotubes extending from GSCs to hub cells. The authors had noted these puncta in their previous publication, but had given them a different role: "Tkv-mCherry localizes along the MT-nanotubes as puncta. Furthermore, using live observation, Tkv-mCherry puncta were observed to move along the MT-nanotubes marked with GFP-αTub (Extended Data Fig. 3d), suggesting that Tkv is transported towards the hub along the MT-nanotubes." Now they show that at least some of the Tkv puncta are in lysosomes in the hub cell, along with the BMP Dpp, and have thus been transferred from GSCs to hub cells and internalized.

Intriguingly, blocking lysosomal activity specifically in the hub cells increases BMP signaling in GSCs relative to that seen in control somatic cyst cells (Fig. 2F). The authors argue that this effect is due to increased availability of Tkv in GSCs, and perhaps also increased Dpp due to the reduced degradation in the hub cells. In other words, **the transfer of Tkv/Dpp from nanotubes into hub cells reduces the levels and range of Dpp signaling from hub cells to GSCs. This is intriguing, but also somewhat opposite to their previous publication, which argued that nanotubes provide a specialized type of contact that increases BMP signal reception in the GSCs.**

**D**

Thank you for raising this important point. Similar concern was raised by multiple reviewers and thus we addressed in above major points (#3). Editor and reviewers’ constructive suggestions made us realize the importance of explaining the apparently contradictory results between current and previous papers. As explained above (#3), we added the text and cartoon to clarify this point.

**The authors do not consider other, less direct mechanisms for their effect- after all, reducing lysosome activity is going to change a lot of things about hub cells, and there are additional BMP signaling components and non-BMP signaling pathways to consider.** I also had problems with the tests they do present.

**C**

This point was also brought up by multiple reviewers and addressed above, where we summarized major changes to this revision (#2).

1) The effects of hub cell-specific lysosome inhibition

**A weakness of the manuscript is that the authors do not directly test whether hub cell-specific inhibition of lysosomes increases Tkv in the GSCs, or Dpp availability. I am concentrating especially on the hub-specific result because it is the only one that is pertinent to their hypothesis, which is not about a general role for degradation, but a specific role of degradation after transfer to hub cells.**

**The only data on Tkv after hub-cell-specific inhibition is the puncta sizes in the hub cell itself (Fig. 2C), with no data on Tkv in the GSCs. And the authors only follow Dpp levels after a manipulation (CQ treatment) that reduces degradation in all of the testes cells, including the hub cells and the GSCs. Not too surprisingly, this increases Dpp levels in the GSCs, but this says little about the specific role of Tkv-Dpp degradation in the hub cells. Expression of a form of Tkv that lacks a ubiquitination site in the GSCs increases Tkv levels and increases BMP signaling in the GSCs, but again this is not surprising.**

**I am also unsure how it would work. The authors provide no evidence that blocking lysosome activity via spin-RNAi or lamp1-RNAi in hub cells reduces Tkv-Dpp internalization into the hub cells; both appear to accumulate in now even larger hub cell vesicles.** **So if Tkv in the GSCs is still being transferred into hub cells, why has BMP signaling in the GSCs increased? If instead the mechanism is via Dpp, now the Dpp is in the hub cell, in a vesicle, bound to Tkv. Somehow its failure to be degraded leads to Dpp's re-release as an active signaling protein?** Additional data and discussion is needed.

**C; text modifications – discussion and clarification**

This concern about Tkv availability in GSC was also raised by multiple reviewers and we addressed above where we summarized major changes to this revision (#1).A concern related to Dpp portion of this comment was addressed below. Please see our response to reviewer 4 (* asterisk indicates the relevant response).

Finally, the authors base their BMP signaling result of Fig. 2F on comparisons with pMad in control somatic cyst cells. Is it possible that what they are seeing is instead a reduction in cyst cell pMad? Are the cyst cells receiving any signaling from the hub cells?

**A, B; address with text, references, or perhaps data**

We added the measurements of absolute pMad levels in cyst cells after inactivation of hub lysosomes (Figure S2A), and also updated all pMad staining images to show a broader area including cyst cells. Relevant portion of the text reads as follows;

“Note that pMad staining in somatic cyst cells (CCs) was unaffected by inactivation of hub lysosomes and could therefore be used to normalize pMad staining intensity (Figure S2A).”

2) The role of MT-nanotubes

**The authors claim that MT-nanotubes that reach from GSC cells to the hub cells promote the internalization of Tkv-Dpp by hub cells. This is certainly a tempting idea, as nanotubes have been invoked elsewhere as mechanisms for molecular transfer between cells. I was surprised, therefore, that the authors provide so little evidence for this, as most of their experiments do not involve changing MT-nanotubes. While they have good evidence that hub cells are internalizing Tkv made by GSCs, they do no provide evidence that the MT-nanotubes play a special role in this process.**

**The only experiment that might provide such evidence comes from Figs 3A and 3B, but here I am relying on my own comparison because neither the text nor the figure legend make any mention of the pertinent data. The panels compare GSC-specific Tkv-GFP overexpression without and with knockdown of osm6, which they previously showed shortens MT-nanotubes. There is, as they published previously, an increase in Tkv on the GSC surfaces, which they had ascribed previously to reduced transfer of Tkv from the cell membrane into the nanotubes. There also appears to be a reduction in Tkv-GFP puncta in the hub cell. However, the authors do not point out either of these results in the main text, and the former result only in the legend. It is difficult to know whether the latter, more important result (reduced hub cell puncta, at least in this image) is real without the kind of quantification they do elsewhere. If they did get this data, however, they would have to be sure that these were lysosomal puncta, as previously they said many of the puncta were those attached to the nanotubes. If you shorten nanotubes, presumably you reduce the Tkv attached to the nanotubes, but that would not say much about transfer from GSCs into hub cells.**

**You will need to explain better in the text and note explicitly what this reviewer noted regarding Figure 3AB, assuming what this reviewer thinks what the figure panels show is accurate.**

We realized that we did not clearly explain original Figure 3A and B (now in Figure 3B, C) regarding the potential role of the MT-nanotube on the Tkv transfer from GSCs to hub cells. As suggested, we scored the number of Tkv positive lysosomes upon inhibition of MT-nanotube formation (Figure 3D). Based on the reduction of Tkv positive lysosomes in hub cells, we propose that the MT-nanotube is involved in Tkv transfer from GSCs to hub cells as expected. We have revised the figure reflecting the new data. Now the relevant result section reads as follows:

“*Intraflagellar transport-B* (*IFT-B*; *oseg2*, *osm6*, and *che-13*) gene products are required for MT-nanotube formation (Figure 3A)^6^. As shown in our previous report, disruption of MT-nanotubes by knocking down *IFT-B* (IFT-KD) causes Tkv receptor retention within GSCs (Figure 3B, C, recapitulation of the previous report)^6^. Therefore, we hypothesized that MT-nanotubes may be required for Tkv transfer from GSCs to hub cells in addition to their function on Dpp ligand reception. To test this hypothesis, we examined the number of Tkv positive lysosomes in the hub. Indeed, we found that inhibition of MT-nanotube formation by knocking down *IFT-B* genes significantly reduced the number of Tkv positive hub lysosomes (Figure 3D). This result, together with the retention of Tkv in the GSC’s cell body suggested that MT-nanotubes may serve as the platform not only for Tkv/Dpp interaction, but also for Tkv internalization from GSCs to hub cells.”

3) The conflicting roles of MT-nanotubes in signaling

**In '15 the authors said that IFT-KD, and thus shortening of the MT-nanotubes, reduced pMad in the GSCs (Inaba et al. Fig. 4), saying this happened because nanotubes were used to carry BMP signaling. However, in this manuscript the authors say that IFT-KD, when coupled with Tkv-GFP overexpression, increased pMad compared with Tkv-GFP overexpression on its own (Fig. 3C,D), presumably because of increased Tkv levels in the GSCs. The authors never discuss the reason for this profound difference in signaling outcomes, and they need to. Both manipulations increase Tkv in the GSC cell bodies. Is it that loss of Tkv trafficking to the hub cells has completely opposite effects depending on Tkv levels (endogenous vs UAS-tkv)? This needs an explanation.**

**D**

This is an important point and was raised by multiple reviewers. We added the text and cartoon to clarify this issue (please see above #3 for detail where we summarized the major points of revision).

4) Mechanisms of transfer

**The authors do not explain or much investigate how Tkv is moving from the GSC cell membrane to the inside the hub cells, and this weakens the manuscript. They do show that a mutant Tkv that cannot be ubiquitinated has reduced transfer to hub cell puncta, so ubiquitination may be part of the transfer process. But this does not get mentioned in the Discussion. The Discussion instead quotes some evidence that similar translocation can occur with other types of receptors in other situations. The authors say that cytonemes can transfer receptors to the signaling cells, but the reference quoted is a long review, and I had trouble finding the data. Could the authors instead quote the primary references?** **It is also noteworthy that here the BMP ligand is not cell-bound, which makes the mechanism of receptor transfer even more puzzling. It might also be worth noting that nanotubes have been invoked in Trogocytosis.**

**You will need to do what is requested by the reviewer here.**

We realize that our overall discussion did not provide ideas about the mechanism of Tkv transfer from GSCs to hub cells. We have added discussion and model cartoon in the revised manuscript to cover this point. Now the relevant part of the discussion read as follows:

“our observation, Tkv transfer between GSCs and hub cells may utilize unique, yet unknown mechanisms. Our data suggest a few clues: A) internalization of Tkv from GSCs to hub cells may not require interaction with Dpp (see Figure 3 model (iii)), B) ubiquitination of Tkv is required for its internalization (the ubiquitination defective mutant of Tkv cannot be internalized), and C) receptor Tkv transferred from GSC does not cause downstream signal activation when it is within hub cells, indicating the possibility that Tkv’s cytoplasmic tail might be facing inside of the transported vesicles (Figure 5K) or the internalized Tkv may be immediately inactivated. Further studies such as the ultra-structure analysis of vesicles and the identification of molecular components will be necessary to elucidate this mechanism.”

**K**, Model. Tkv expressed in GSCs is trafficked to MT-nanotubes then internalized into hub cells with its ligand Dpp. Tkv/Dpp containing vesicles fuse with hub lysosomes and subjected to degradation.

Please note that our description about cytoneme was not accurate. We mistakenly stated that the ligand-receptor complex is taken up by signal sending and recipient cells. We apologize for the confusion. We corrected the description as follows and cited the original reference:

“Cytonemes, another type of signaling protrusion^24^ ^25^, traffic non-membrane-bound ligands from signal sending cells to signal receiving cells that are located in distance each other. Although cytoneme-derived ligands are internalized into the receptor expressing cells^26^, the opposite instance, namely receptor internalization into ligand producing cells, has never been reported.”

**It might be helpful to note somewhere what part of the Tkv protein was tagged in their three different Tkv constructs, as the reader may wonder whether the transfer is of all of Tkv (ECD and ICD), or only fragments. While the protein trap is tagged in the ECD (and is not entirely normal- look at homozygous wings), as far as I can figure out the Kornberg constructs are both tagged at the C terminus and thus the ICD.**

**You will need to provide this information.**

Thank you for this suggestion. We added the information of each Tkv construct in the text. Whereas it is a sound concern that differently tagged proteins may behave differently, based on two observations, we consider these constructs are equivalent and reliably report the endogenous protein’s behavior: 1) we observed the signal of C terminus tagged Tkv (mCherry) from Kornberg’s lab entirely colocalizing with Tkv GFP-trap (N terminus tagged), indicating that both constructs likely represent the localization of full length Tkv protein (FigureS1). 2) we did not detect any phenotype in the testes of homozygous protein trap flies (Tkv-GFP-trap).

We added information in Methods section, and also softened our claims to acknowledge the caveats (such as truncation of the tag portion and localization/stability change).

The relevant portion in Methods section read as follows:

**“Verification of the functionality of fluorescently tagged proteins**

We acknowledge the general problems with fluorescently tagged proteins, such as truncation of the tag portion, mislocalization/aggregation and stability change. In addition, Gal4/UAS mediated expression may not accurately reflect endogenous protein amount. Moreover, as we noted in our previous report7, we observe that mCherry but not GFP itself is trafficked from GSC to hub cells, indicating the possibility that mCherry tag may promote the trafficking regardless of the transgene. However, we had to largely rely on fluorescently tagged proteins and live observation to determine their localization and behavior. This was due to technical difficulties caused by low levels of endogenous protein amounts and/or difficulties to preserve structure during fixation. Thus, we summarized the functional verification of constructs used in this study here.

The Dpp-mCherry knock-in fly and Tkv-GFPtrap fly are both homozygous viable and we did not detect any phenotype in the testis, and we observed almost complete colocalization in hub area (Figure S1B). The complete colocalization of nosGal4 driven expression of Tkv-mCherry (C terminus tagged) with Tkv-GFPtrap (N terminus tagged) indicates that both constructs likely represent the localization of full length Tkv protein (Figure S1C). Moreover, the complete colocalization of Tkv-mCherry (C terminus tagged) with Tkv-GFPtrap (N terminus tagged) indicates that both constructs likely represent the localization of full length Tkv protein (Figure S1C). In summary, we consider these constructs to be equivalent and to reliably report an endogenous protein’s behavior. Nevertheless, future studies using alternative methods might be necessary to verify the results obtained from these constructs.”

__________

reviewer 2

Building off of their previous work in identifying the importance of microtubule-based nanotubes to ensure close-range BMP signaling within the testis niche, the authors provide compelling evidence for hub cell-dependent lysosomal degradation of BMP ligand-receptor complexes in ensuring restriction of this niche signaling exclusively to the germline stem cell (GSC) population. The experiments presented throughout the manuscript are well-designed and the data (particularly photobleaching of ectopically diffusing dpp) is quite convincing. Overall, the work is a logical and thoughtful continuation of the original nanotube manuscript. The only difficulty with this work is in the author's interpretation of some of the data; specifically a lack of discussion regarding some surprising results.

Major issues:

While some experiments (particularly labeling somatic cell membranes in some genetic backgrounds) would be helpful in addressing these concerns the vast majority could likely be addressed by expanding upon the discussion.

**A major deficit in the interpretation of results in this manuscript is a lack of discussion about somatic cyst stem cells and cyst cells and the potential role they may be playing. It is well established that CySCs also produce dpp (and gbb) ligands and thus could easily also be signaling to GSCs. Particularly given the lack of staining for somatic cells throughout the results it is difficult to know how seriously the role for somatic cells should be considered.**

**B**

This important point was raised by multiple reviewers, we addressed the involvement of somatic cyst stem cells above in the section ‘major points of revision (#2)’.

In addition, we revised all pMad staining image to include the cyst cells and also show that the pMad level in cyst cells was unchanged after inactivation of hub lysosomes (Please see detail in the response to the reviewer1’s comment regarding the role of cyst cells)

**Discussion of results regarding the link between hub-based lysosomal degradation of GSC-derived Tkv and an increase in pMAD staining/BMP signaling within GSCs. The data is all compelling but some confusing disparities need to be addressed. It is unclear how Tkv internalized in hub cells and associated with (nonfunctional) lysosomes would still be capable of transducing dpp signals to increase pMad in the GSCs. Alternative hypotheses (such as Tkv on the cortex signaling extensively and no longer being trafficked to the hub cells) should be discussed. Additionally, if Tkv is incapable of being degraded this should not prevent its localization to MT nanotubes—yet the authors find a signficant decrease in Tkv localized to hub cells (Which at this resolution is presumably what you would expect to see).** Clarification on this point would be helpful and could, potentially, compellingly expand upon the author's model.

**C**

Thank you for raising this critical point. Similar issues were brought up by multiple reviewers. We summarized the major changes above in point #1.

**Related to Figure 4: It is difficult to know from the images shown that the increase in Dpp is on the germ cell membrane rather than also in somatic cells. As increased BMP signaling in the CySCs is known to cause defects (increased proliferation, competing with GSCs for niche occupancy; Lu et al, 2019) it would be helpful to know if BMP is indeed also increased within the somatic stem cells and their daughters. In addition, it is known that Jak/STAT signaling from the niche increases dpp expression in CySCs. It would be helpful for the authors to show that their manipulations of lysosome function within the hub is not also disrupting Jak/STAT and causing complicated downstream effects that need to be taken into account in addition to direct changes to BMP signaling occurring through loss of lysosomes in the hub.**

**A, B**

This point was also brought up by multiple reviewers and addressed above, where we summarized major changes to this revision (#2).

Figure 4B arrowheads—it's not possible to know that these are germ cells without some type of counterstaining as they could just as easily be somatic cells. This is a small point speaking to a larger "issue" of somatic involvement in any of these events being completely ignored. This becomes more significant when thinking about mechanism—in all images of lysotracker (and similar), the germ cells appear to have notably fewer lysosomes than the hub. Is this true? And if so, does this contribute to the substantial increase in dpp signaling distant from the hub (as germ cells would not be able to degrade active Tkv/dpp complexes on their own)? If the dpp puncta associated with lysosomes distant from the hub in figure 4 are, in fact, within somatic cells rather than germ cells, it would bolster this particular interpretation. The reverse (that the puncta are in fact within germ cells) gives further credence to the author's preferred interpretation. Either way, having this information would make the conclusions more convincing.

**B**

As suggested by this reviewer, we have conducted the live-imaging experiments to determine the cell types that are receiving diffusing Dpp ligand. The new experiment revealed that the Dpp positive lysosomes appear both in the cell body of somatic and germ cells after chloroquine treatment. Importantly, we barely observed Dpp positive lysosomes in GSCs, further confirming the idea that GSC does not digest Dpp/Tkv complex within its cell body. We added the data in Figure5E (see below).

**E,** A representative image of the testis tip of *dpp-mCherry* knock-in after 4-hour incubation with CQ. Blue dotted lines outline the hub. Yellow lines outline GSCs. Red; Dpp-mCherry, Green; Chd64-GFP (CB03690, a CySC marker) and FM1-43, membrane dye. Right panels (1-4) show high magnification images of the indicated cell types.

We would like to note that we usually detect roughly same number of lysosomes in each cell types. We consider that the seemingly more signal of lysotracker in the hub area pointed out by this reviewer is caused by the cell size difference between hub cells and germ cells.

Minor issues:

**In Figure 1 (and for many other figures) the different cell types are not adequately indicated. Particularly for those reading this paper who do not work in this field, I think it would be quite challenging to identify where hub cells versus GSCs are present in these images. Outlines similar to those utilized in other panels of figures could be helpful, though counterstaining with a hub cell marker would be ideal.**

**Outlines would be helpful if you don’t already have counterstain data**

Thank you for this suggestion. We added outlines that encircle hub (and GSCs, if possible) to most of our images. When an image shows only a portion of the hub, we added a diagram next to the image to help readers comprehend the overarching anatomy.

Note: Clonal expression of Tkv-mCherry. Right two GSCs (arrowheads), but not other GSCs are expressing Tkv-mCherry transgene.

**Tkv-mCherry localizes significantly to GSC cell membranes in addition to its accumulation within hub cells (Fig1 O,N). Is this receptor on the cortex non-functional? In addition, the image of a testis 3days post-heat shock appears to have less Tkv localized to GSC membranes. Is this just in this particular image or is a movement from cortex to nanotubes a feature of Tkv signaling in GSCs?**

**Clarification in text or legend is sufficient**

Tkv-mCherry on the cortex seen at day1 after heat shock should be functional as we observed high pMad staining in this time point (see right).

Tkv localization change during the time course experiment, we clarified these points in the text as follows:

“When expression of Tkv-mCherry was initiated by heat shock (*hs-flp, nos-FRT-stop-FRT-Gal4, UAS-GFP, UAS-tkv-mCherry*), Tkv-mCherry was first observed on the GSC plasma membrane and in the GSC cytoplasm as puncta (day 1 after heat-shock). After 2 days post heat shock, its signal in the hub became evident. Finally, after 3 days, the Tkv signal along the GSC plasma membrane was reduced and the Tkv signal in the hub increased further (Figure 1D-F). These results indicate that Tkv seen in hub area is originated from GSCs.”

___________

reviewer 3

Comments to Authors:

In this manuscript, the authors used a series of genetics and cell biology tools in fly testes to test a model that Tkv, the receptor of the BMP signaling pathway, is generated by germ stem cells, then transport to niche cells, where excess Tkv gets degraded by lysosome. Later in the manuscript, the authors propose that the ligand, Dpp, is also regulated similarly. The advantage for the niche cell, instead of germ cell, is to degrade the Dpp-Tkv compound, in order to attenuate the excessive BMP signaling. As the authors pointed out in the Discussion, similar context-dependent regulation of signaling pathways were reported in other systems, therefore the novelty could be the finding in this system, i.e. communication between Drosophila germ cells and hub cells.

There are a few major questions that should be considered:

-**To study degradation pathways of the endogenous protein, experiments using antibodies that recognize the corresponding endogenous protein would be a lot more reliable than using tagged version, this is because most of the fluorescent protein tag is quite stable and could result in accumulation of proteins. I have some suggestions here: (1) It would be the best if experiments can be done using antibody that recognizes the endogenous protein. (2) If such a reagent is impossible, I feel these experiments should be done using less stable FP tag such as unstable GFP variants. (3) Experiments could be done and the results are compared under the condition that the Tkv level is compromised. For example, the GFP tagged Tkv could be studied when one copy of endogenous tkv is removed using heterozygotes, and RT-PCR or immnunoblot could be performed to compared the overall levels. (4) A control immnunoblot should reveal whether detected GFP or mCherry is indeed for the fusion protein (Tkv-GFP and Dpp-mCherry). (5) Overexpression of GFP or mCherry by itself could be another control. I feel some of the above suggested experiments should be done to strengthen the point of Tkv/Dpp being degraded by lysosome in hub cells to restrict only stem cells receive this signaling, which is major conclusion for this manuscript.**

**A, B. The reviewer’s thoughts are valid; however, this may be too much to request for a Short Report. If you have a fusion with a less stable FP, we encourage you to add the data. Alternatively, you could add any data that can address the issue raised by the reviewer and acknowledge the caveat in the text; you should also soften the text about the model.**

Thank you for raising this issue and suggestions. A similar point was raised by reviewer #1 and we discussed it above (please see the response to reviewer #1’s last comment). In addition, as we noted in our previous report (Inaba et al., Nature 2015), we observe that mCherry but not GFP itself is trafficked into hub cells. Therefore, we confirmed the data obtained from each particular construct by using a different tag.

We added this information in the revised text. We also acknowledged the caveats (such as truncation of the tag portion and localization/stability change) and softened our model (Please see our response to reviwer1’s last comment).

- **The paper used pMad as a readout of the BMP signaling, which should be enriched in the stem cells, but the immunostaining in the control samples showed very minimal enrichment, if any, in the controls, such as in Figure 2G and 3E. To determine the differentiation state of the germ cells, if pMad staining has caveats, why not using other markers, such as Stat and markers for spectrosome/fusome, etc.?**

**B. Please add these data if you already have them.**

We added the image of STAT92E staining in the revised Figure S2C-E.

- **In Figure 2J, since the Tkv-S238A-GFP is driven by germ cell driver, it is not surprising to see changed localization in the germline stem cells. Here, the overexpression is a concern and such an experiment should be performed by making this mutation at the endogenous gene.**

**Please add the data if you already have or note the caveat in the text if you haven’t done the experiment.**

Our comparison between Tkv-S238A-GFP and Tkv-GFP (both under the regulation of a UAS promoter) was performed by using the same germ cell driver (nosGal4). We realized the description was unclear in our original manuscript. We added the information in the main text as follows:

“We found that Tkv-S238A-GFP, in which the serine residue required for Smurf-dependent ubiquitination was mutated, exhibits striking differences from wild type Tkv-GFP on two accounts. When both constructs were expressed in GSCs (*nosGal4>tkv-GFP vs. nosGal4>tkv S238A-GFP*), the amount of S238A-Tkv protein was significantly higher than that of wild type Tkv (Figure 4A, B).”

We would like to note that Tkv-S238A knock-in is anticipated to be heterozygous lethal (i.e. dominantly lethal) due to its degradation defective nature. We acknowledged the caveat about possible overexpression by using Gal4/UAS system in the text.

“We would like to note that the Gal4/UAS mediated expression may not accurately reflect endogenous protein amount.”

- **The paper does not address the origin and regulation of other BMP signal such as Gbb. Both Gbb and Dpp can activate BMP signaling. This is a minor point and would be good to know.**

**B**

We agree with this reviewer that we must consider other BMP ligands. Indeed, Gbb and Dpp are both reported to bind to Tkv and cooperatively maintain GSCs in Drosophila testis. However, Gbb is broadly expressed in somatic cells outside of the niche, whereas Dpp is concentrated in the hub (Kawase et al., Development 2004). Therefore, we focused on Dpp in this study because our primary purpose is to examine the regulation of short range signaling. We added this information in the revised manuscript, which read as follows:

“It should be noted that the other BMP ligand, Glass bottom boat (Gbb) is also known to bind to Tkv and cooperatively maintain GSCs. Gbb is broadly expressed in somatic cells outside of the niche, whereas Dpp is concentrated in the hub^20^. Therefore, we focused on Dpp to examine the regulation of short-range signaling.”

- **On page 5, this sentence in "neither downregulating hub lysosome (spin RNAi) nor Tkv trafficking to the hub (IFT-KD), both of which are expected to compromise Dpp-Tkv degradation in the hub, were not enough to impact differentiation." is confusing, by making a double negative, do the authors mean that these conditions were enough to impact differentiation? Then, the next sentence "However, we found that compromising Tkv trafficking to the hub (IFT-KD) combined with overexpression of Tkv (TkvOE) (referred to as IFT-KD/Tkv-OE) showed prolonged pMad staining in SG populations". However, these two combined conditions: IFT-KD + Tkv-OE is different from the previous two conditions: spin RNAi or IFT-KD, I do not quite get the points for this paragraph of comparing different conditions.**

**We agree with this reviewer and would like to ask you to reword the text.**

We revised the text to correctly state these conclusions, which now reads as follows:

“Although shutting down the Dpp signal is required for proper differentiation, neither downregulating Tkv trafficking to the hub (*nosGal4>IFT-B RNAi,* referred to as IFT-KD) nor Tkv overexpression (*nosGal4>tkv,* referred to as Tkv-OE), both of which are expected to increase the available Tkv in GSCs, were alone found to impact differentiation.”

__________

reviewer 4

"Niche cell lysosomes self-restrict the signaling via receptor-ligand degradation" submitted to PLoS Biology by Ladyzhets S et al.

There is increasing amount of evidence showing that niche-derived molecules function over a short range on resident stem cells to promote their self-renewal, while differentiating daughters are shielded from such stemness-promoting signals. Inaba M demonstrated previously that in Drosophila testis, GSCs generate a novel type of microtubule-based nanotubes, which allows them to sense niche-derived stemness promoting ligand Dpp by transporting its receptor Tkv along these nanotubes and into the hub cell cluster. In this manuscript, the authors find that Tkv, in addition to its association with nanotubes, also localize within hub cells and colocalizes with lysosomal markers. They go on to show that these Tkv puncta are derived from GSCs and sensitive to the disruption of lysosomal function in hub cells or to lysosomal drug treatment. Interestingly, another Dpp receptor Put and hub-derived Dpp also co-localize with these lysosomal makers and signaling activation reporter TIFP is also activated in these lysosomes. Compromising lysosomal activity in hub cells results in enlarged Tkv puncta in hub cells and leads to detectable Dpp signaling activation in GBs, which is not normally detected in wt testis. They further show that Tkv.S238A, a Tkv variant resistant to Smurf-mediated ubiquitination, is accumulated on GSC membrane and less detected in hub cells (on nanotubes). Lastly, using testis in vitro culture and lysosomal inhibitor treatment, the authors show that Dpp is detected outside the hub cells. Based on these data, the authors proposed that these GSC-derived nanotubes, in addition to allow GSCs to sense niche-derived Dpp, also serve another purpose - to limit the signaling range of Dpp by means of degradation Dpp/Tkv complex.

Overall, this is an interesting manuscript which addresses the mechanism of how the niche activity is spatially regulated in the Drosophila male GSC niche and is of general interest for the readership of PLoS Biology. Following experiments, if confirmed, should be able to improve/strengthen the conclusion present in this manuscript.

Main concerns:

**The authors propose the following model: GSC-produced Tkv is transported along the MT-nanotubes into the hub region at the interface between GSC and hub cells; Tkv binds hub cell-produced Dpp and initiates downstream signaling in GSCs; the Tkv/Dpp complex is subsequently translocated from GSC surface into hub cells and degraded in hub cells via lysosome-dependent activity to limit Dpp signaling range. The data present here show that GSC-derived Tkv is transported to the hub region (Fig. 1) but no further evidence is provided to judge its cellular localization. It is not clear whether these Tkv puncta are present at extracellular space between hub cells or they are (endocytosed to) inside the hub cells. This is one of the key claims in this manuscript. To sustain their model, the authors should provide unambiguous evidence to show that Tkv puncta are indeed present inside hub cells and colocalized with hub cell-derived lysosomes. One potential approach is to conduct TEM analysis for their subcellular localization. Furthermore, if these Tkv puncta are indeed inside the hub cells via an endocytosis-mediated process, the intermediate Clathrin-bound endocytosed vesicle could also be detected within the hub cells (also see next point). If confirmed, these results will greatly support the proposed model.**

**B**

We agree with this reviewer that the localization of Tkv to hub lysosomes is the major claim of our manuscript and thus must be thoroughly characterized. We added additional imaging data by visualizing the hub cell membrane and analyzed localization of Tkv by Imaris software. We concluded that Tkv is mainly localized inside of hub cells (Figure 1B, see below).

**B,** A representative image of a hub and surrounding GSCs expressing Tkv-mCherry (red) and GFP (green). Plasma membranes of cells are visualized by FM1-43 dye (green). Tkv expressed in GSCs is seen in the hub cells. A testis from *hs-flp, nos-FRT-stop-FRT-Gal4, UAS-tkv-mCherry, UAS-GFP* fly was imaged 2-days after heat shock. **B’** A graphic interpretation of the image **B.** Blue box indicates the area used for three-dimensional rendering in **B”**; **B”** A three-dimensional rendering image of the hub area (blue box in **B’**).

The imported vesicles may contain both of GSC and hub cell membrane. We have been trying immunogold staining but have not succeeded in determining Tkv localization within hub cells due to technical issues.

**According to the proposed model, lysosomal activity in hub cells is essential for the degradation of the Tkv/Dpp complex and thus limits Dpp functional range. Some key supporting data include 1) enhanced Dpp signaling in germ cells when hub cell lysosomal activity is compromised (Fig. 2); 2) no alteration in dpp transcription under this condition (Fig. S2); and 3) Dpp can be detected outside the hub cells under in vitro culture condition in the present of lysosomal inhibitor (Fig. 4). Several concerns need to be clarified. Firstly, if the Tkv/Dpp complex is located within the hub cells as proposed by the authors, inhibition of lysosomal activity in hub cells would lead to defects in the degradation of the Tkv/Dpp complex inside the hub cells. How could it lead to expanded Dpp activity range in germ cells? Does it mean the Dpp/Tkv complex undergoes exocytosis to be secreted out of hub cells, Dpp is dissociated/released from Tkv and diffuses outside the hub to functions again?**

**C**

This important point was raised by multiple reviewers. We addressed this point with new data and summarized major changes above (#1).

Regarding the Dpp portion of this comment, we have removed the previous statement. Please see our response below about the Dpp diffusion (* asterisk indicates the relevant response).

**It is possible that disruption of lysosomal activity may affect other hub-derived signals. Have the authors checked the expression and activity of other signaling pathways such as the JAK/STAT signaling activity which could activate Dpp signaling upon ectopic expression. Secondly, dpp expression is of importance to the model. Although Dpp in situ data provides a qualitative measurement, quantitative measure is warranted. qPCR should be conducted to confirm dpp transcription level.**

**A**

This point was raised by multiple reviewers. We tested the effect of inactivation of hub lysosomes on the JAK-STAT pathway and added the data and description (#2).

Regarding this reviewer’s concern about the quantification of Dpp *in situ* analysis, we reanalyzed our data using particle counting function of “octane” plugin (https://github.com/jiyuuchc/Octane), for Fiji which collectively provide decently accurate information about the expression level. In support of this notion, we can detect the reduction in Dpp FISH signal upon knockdown of Dpp (FigureS4 DppRNAi 29°C). Using the new method, we obtained results similar to our old measurement. We updated the graph in Figure S4.

**It was shown in their 2015 Nature paper, disruption of MT-nanotube by IFT-KO reduces pMad signaling in GSCs without noticeable upregulation in GBs, suggesting diffusion of Dpp outside hub cells may not necessary induce signaling activation.**

**D; relates to item above**

* The concern about our statement about Dpp diffusion was raised by multiple reviewers. We understand that our previous statement that ‘Removal of MT-nanotube or inhibition of hub lysosomes increase the amount of Dpp diffusion’ was not sufficiently supported by the data. Moreover, we realized that increased Tkv amount can solely enhance the signal without changing Dpp amount because Tkv S238A (which cannot be transferred to hub lysosomes) expression increases GSC’s pMad levels. We revised overall text to downplay our statement. Now the relevant part is the “results” section entitled:

“**Dpp ligand produced in hub cells diffuses outside of the hub”**

.

**Thirdly, FRAP recovery rate in Fig. 4 seems to be extremely fast with a full recovery around one minute. In L3 imaginal disc where Dpp is expressed at a much higher level along the anterior/posterior boundary compared to that in the hub cells, the FRAP recovery rate for a column of cells of 10um width (the diameter used in this study) right next to this boundary is more than 10 min (Kicheva A et al., Science 2007, Zhou S et al., CB 2012). It is possible in vitro CQ-treatment resulting in some background of this set of experiments. Indeed, the non-specific background is increased in these testes. A control experiment should be conducted to exclude this possibility.**

**Please add requested control and explanation in text.**

Thank you for pointing these issues out. First, we found a mistake on the time scale of our FRAP experiments in the original figure. Although the time lapse imaging was taken every 10 seconds, it was shown as taken every second. Thus, the actual diffusion speed is 10 times slower. We apologize for this error. We corrected the graphs accordingly. The diffusion speed measured in our FRAP experiment is now comparable to the report of in Zhou S et al., CB 2012. We have cited their report and added the discussion about the possible mode of diffusion observed in our experiment.

Second, as pointed out by this reviewer, we realized that the chloroquine treatment slightly increases the background fluorescent level in our FRAP experiment. For more accuracy, we tried the experiments without chloroquine treatment. Notably, this new setting resulted in a similar recovery curve to the original experiment but with reduced data variation. We revised the figure using the new data (Figure5F-I) and also added the result of control experiment (Figure5J and FigureS5).

**As discussed in their early paper (****Inaba M et al., Nature 2015), the microtubule-nanotubes are very sensitive to fixation. It is surprised to note that the fixing method used in this manuscript does not aim to stabilize microtubules for nanotubes detection and Tkv co-localization (see Methods). It is possible that even under their previously described fixation condition with addition of a low concentration of microtubule-stabilizing drug Taxol, not all MT-nanotubes are preserved and detected at a confocal resolution due to its dynamic nature. The fixation method used in this manuscript likely disrupts MT-nanotubes and results in MT remnants. The authors should provide evidence to demonstrate that these Tkv puncta do not associate with remnants of disrupted MT-nanotubes during fixing process.**

**D; you will need to address this important concern satisfactorily**

Most of the experiments were conducted by using live tissue without fixation, therefore, the method to preserve MT-nanotubes was not necessary. The only experiments conducted with fixed samples were the pMad quantification and tumor formation scoring, neither of which needed MT-nanotube preservation for scoring purposes. We realized that the method of each experiment was not clearly stated. We added the information in the figure legends as well as in the methods section.

**Although some contact-dependent signaling events involving exchanges of molecules between ligand-sending and receptor-producing cells have been documented, including cytonemes in fly and Trogocytosis in vertebrate systems, these ligands (in the case of receptor endocytosed into ligand-sending cells) are membrane-bound proteins. Since Dpp is produced in the hub cells and believed to be secreted into extracellular matrix, how can extracellular Dpp bind Tkv to activate downstream signaling in GSCs then "extract" transmembrane receptor Tkv from GSCs and transfer it into hub cells for degradation? If proved, this is an intriguing piece of data. Since signaling sensor TIPF is activated in these lysosomal compartments, why downstream signaling (pMad or Dad-lacZ) is not detected in these hub cells under this circumstance? Note that the authors refer this reporter as Dpp signaling activation in other contexts including those CQ-treated samples (Fig. 4).**

**B**

This is a legitimate concern and raised by multiple reviewers. We hope this point was partially addressed above in major point #1.

Although the mechanisms of Tkv transfer from GSC to hub cell remains elusive, our data suggest a few clues. We added the discussion with a cartoon referring these points in the revised manuscript. Please see our response for reviewer1, 4).

**The novel function of MT-nanotubes described here is similar to a recent study showing that Drosophila female GSCs deploy an Actin- and MT-dependent "cytosensor" to sense and limit niche-derived Dpp molecule (Wilcockson SG and Ashe HL, Dev Cell 2019). This paper should be cited and discussed in this context.**

**Please add the citation and a brief discussion.**

We added the discussion and citation, which reads as following:

*“Drosophila* ovary have also been reported to project cellular protrusions into the niche cell cluster to sense and access a reservoir of Dpp^30^. Intriguingly, these projections are also required for attenuation of Dpp signaling, suggesting the possibility that female GSCs utilize a similar mechanism to degrade signaling components.”

**Page 6, line 2, "…, how can IFT knockdown cause a tumor located outside of the niche?". This is an inaccurate statement. The authors show in page 5, 3rd paragraph that "… nor Tkv trafficking to the hub (IFT-KD), both of which …, were not enough to impact differentiation." The condition mentioned here should be "IFT-KD/Tkv-OE".**

**Please make this minor correction.**

We agree that this statement was not accurate. Based on the suggestion from this and other reviewers and editor, we removed the portion proposing the idea that increased Dpp is the cause of the tumor. Thus, this statement was also removed.

Please also see our response above (* asterisk indicates the relevant response).

**Page 5, paragraph 4, the statement "… tumor formation is likely caused by a defect within GSC…" is not well supported by data presented herein. The germline tumor is observed in IFT-KO/Tkv-OE driven by nos-Gal4 but not bam-Gal4. Firstly, it's well known that nos-Gal4 is expressed highly in germline than bam-Gal4. Secondly and importantly, nos-Gal4-mediated IFT knockdown disrupts MT-nanotubes, thus potential releasing Dpp from the hub cells, while bam-Gal4-mediated IFT knockdown does not disrupt the distribution of hub cell-derived Dpp. Thus this data need to be interpreted cautiously. The elevated Dpp signaling activation in IFT-KO/Tkv-OE could be a combinatory effect of affecting microtubules (by IFT-KO) and spontaneous Dpp signaling activation (by Tkv-OE), not necessary a result of Dpp diffusion. Can the authors distinguish this from their model?**

**We agree with the reviewer, and suggest that the authors downplay and shorten this part of the speculation.**

We understand that our previous statement that ‘Removal of MT-nanotube increases Dpp diffusion’ was not sufficiently supported by the data. We have edited the overall statement about Dpp diffusion to downplay our statement. Please also see our response above (* asterisk indicates the relevant response).

References

Kawase, E., Wong, M. D., Ding, B. C. & Xie, T. Gbb/Bmp signaling is essential for maintaining germline stem cells and for repressing bam transcription in the Drosophila testis. Development 131, 1365–75 (2004).

Inaba, M., Buszczak, M. & Yamashita, Y. M. Nanotubes mediate niche-stem-cell signalling in the Drosophila testis. Nature 523, 329–32 (2015).
