# Supplementary material for: Self-limiting stem-cell niche signaling through degradation of a stem-cell receptor
Source: PLoS Biol. 2020 Dec 14;18(12):e3001003. doi: 10.1371/journal.pbio.3001003 (PMC7769618; doi:10.1371/journal.pbio.3001003)
Supplement: Attachment — Submitted filename: plos B revision2 response.docx [file pbio.3001003.s014.docx]

Point by point responses;

Editor comments are in **Red**. Reviewer comments are in **black**. Our responses are in **blue**.

Dear Dr Inaba,

Thank you very much for submitting a revised version of your manuscript "Stem-cell niche limits its signal via degradation of stem-cell derived receptor" for consideration as a Short Report at PLOS Biology. Thank you also for your patience as we completed our editorial process, and please accept my apologies for the delay in providing you with our decision. This revised version of your manuscript has been evaluated by the PLOS Biology editors, the Academic Editor and two of the original reviewers.

The reviews are attached below. You will see that the reviewers find the manuscript very much improved and Reviewer 2 is now satisfied. However, Reviewer 1 has raised several points that remain to be addressed. After discussing the reviews with the academic editor, we encourage you to address all the points. While both points 2 and 4 can be addressed textually, we would like you to perform the experiments suggested in points 1 and 3.

In light of the reviews, we are pleased to offer you the opportunity to address the remaining points from the reviewers in a revised version that we anticipate should not take you very long. We will then assess your revised manuscript and your response to the reviewers' comments and we may consult the reviewers again.

We expect to receive your revised manuscript within 1 month.

Please email us (plosbiology@plos.org) if you have any questions or concerns, or would like to request an extension. At this stage, your manuscript remains formally under active consideration at our journal; please notify us by email if you do not intend to submit a revision so that we may end consideration of the manuscript at PLOS Biology.

Thank you again for your submission to our journal. We hope that our editorial process has been constructive thus far, and we welcome your feedback at any time. Please don't hesitate to contact us if you have any questions or comments.

Sincerely,

Ines
--
Ines Alvarez-Garcia, PhD,
Senior Editor,
ialvarez-garcia@plos.org,
PLOS Biology

We would like to again thank the editors and reviewers for their careful reading of our original and revised manuscript for further providing us with important points that were still missing from our previous version. In particular, we fully agree that some previously published results needed be discussed more thoroughly, and that possible mechanisms should be proposed. We reworked further on the manuscript. We also provide new data to address additional points.

We moved the figure showing the results of lysosomal inhibition and Tkv ubiquitination to later in the manuscript, as these results both provide additional clues about the mechanism. We also worked on the discussion added an example of ciliary ectosome transfer to neighboring cells.

As you see in our response below, we essentially addressed all comments and concerns newly raised by a reviewer.

--------------------------------------------------
Reviewers’ comments

Rev. 1:
The manuscript has been extensively reworked, with additional data and some changed interpretations. Most notably, the authors now avoid discussing any role for changes in the release of Dpp as an explanation for the increased BMP signaling seen after inhibition of either lysosomal function in the hub cells, or reduction of MT-nanotubes and increased Tkv in the GSCs. Instead, the authors suggest that these effects are caused by increased levels of Tkv in the GSCs caused by reduced transfer to the hub cells.

1) Threads- The authors now provide evidence that loss of lysosomal function, either generally through CQ treatment, or in the hub through RNAi of spin or lamp1, causes an increase not only in Tkv-GFP-trap in round lysosomal puncta in the hub, but also in fainter threads. They interpret these as "likely" being Tkv along MT-nanotubes, and further suggest that much of the Tkv is on the MT-nanotube (GSC) membrane, where it would allow the GSC to receive more Dpp signaling and thus increase pMad.

However, the authors do not co-localize threads with MT-nanotubes, saying there are technical difficulties, and this greatly weakens their argument. I can understand the difficulties in the case of upd-gal4-driven RNAi, since they cannot combine this with the nos-gal4 they usually use to drive MT-nanotube markers. However, there is no reason they could not use nos-gal4-driven MT-nanotube markers along with tkv-GFP-trap, and then treat with CQ to accentuate the threads. In ref 6 they also visualize nanotubes with antiserum against Klp10A, something they could couple with upd-driven RNAi.

We understand the importance of colocalizing MT-nanotubes and Tkv threads.

We have now succeeded in visualizing colocalization of MT-nanotubes and Tkv thread in the testis expressing αTub-GFP and Tkv mCherry together under the nosGal4 driver. We also scored the frequency with which MT-nanotubes are decorated by Tkv with or without CQ treatment. These new figures are added as new Figure 3L and M, below). As suggested, we initially tried anti Klp10A staining together with Tkv-GFP-trap with or without Spin RNAi. However, we could not preserve the Tkv-GFP signal within the hub after the fixation and staining process.

2) Mechanism?- The authors still do no explain why inhibition of Tkv degradation inside hub cells would lead to higher Tkv on the surface of the GSCs. In order for this mechanism to work, blocking lysosomal activity has to somehow reduce the transfer of Tkv from GSCs into hub cells. Once the Tkv is transferred into a hub cell it is no longer functional in the GSC, and whether or not it is degraded makes no difference to the GSC. If transfer follows the model in Fig. 5K, why would having Tkv in non-functional lysosomes reduce the hub cells' endocytosis of Tkv-containing exosomes? Or does blocking lysosomal function generally reduce endocytosis? The authors have to supply a plausible mechanism, even if it is hypothetical.

We understand that the data in which inhibition of lysosomes resulted in decreased Tkv endocytosis needs explanation. As pointed out, upon inhibition of lysosomes in hub cells, we observed higher Tkv on the GSC surface (nanotubes) in addition to enlarged Tkv positive lysosome in hub cells. Impairment of degradation of endocytosed Tkv has been reported in hrs mutant in Drosophila ovary. Similar to our case, hrs mutant causes enlarged endosomes AND increased Tkv on the cell surface (Jekely et al., 2003). Therefore, we speculate that defective of degradation of endocytosed Tkv may initiate feedback mechanism to prevent further endocytosis. We have added discussion about this point in the manuscript which reads as follows:

“It is still unclear how a lysosomal defect causes increased amounts of Tkv on MT-nanotubes. Similar to this case, it has been reported that a degradation defect of endocytosed Tkv subsequently increases the Tkv protein on the plasma membrane [20], suggesting that there is a potential feedback mechanism such that a lysosomal defect inhibits further endocytosis in the cells, leading to increased Tkv on the cell membrane and signal hyperactivation.”

3) Indirect mechanisms- These difficulties led the reviewers to suggest less direct mechanisms for the lysosomal effects on BMP signaling in the GSCs, for instance by changing BMP (Dpp and Gbb) production in hub cells or cyst cells, or via some less direct effect through the hub cells' production of JAK ligands. The authors have added some evidence, but it could be more complete, and leaves some open questions.

They show that that Dpp mRNA levels are similar in affected hub cells, but do not say much about Dpp release or diffusion. They were unable to detect any Dpp mRNA in normal or experimental cyst cells, despite published evidence that they do make it, making the negative data difficult to assess. They did not examine Gbb in hub or cyst cells; aside from in situs, there are some Gbb-GFP lines at Bloomington that might work in the testes. The authors do show that early cyst cell development seem unaffected by hub cell-specific reduction of lysosomal activity, as visualized with Zfh-1.

However, while the authors do not see any effect in nuclear STAT, they do see a profound effect on cytoplasmic STAT staining in germ line cells quite distant from the hub (S2F vs G). The authors suggest that the increased BMP signaling has inhibited differentiation of GSC daughter cells and that this maintains high cytoplasmic STAT. But the authors need to provide some evidence for failed differentiation (Bam, as they did after IFT-KD?). And is failed differentiation known to maintain high STAT levels?

We thank this reviewer for this comment. We fully agree that we did not have explanation as to why we could not detect dpp transcript in CySCs even though evidence is provided by previous publications. Moreover, we did not explain why the STAT92E level becomes high in the cells outside of the niche upon inhibition of hub lysosomes. Additional consideration of these points is described below.

i) Dpp levels

Our original manuscript included the suggestion that the amount of functional Dpp protein released into the testis from the hub might increase when MT-nanotubes are not present and Dpp, together with Tkv, might be involved in elevation of pMad levels in GSCs. However, the reviewers recommended we remove this statement because we did not have sufficient evidence to show an increase in Dpp protein. In addition, we subsequently found that there were no detectable changes Dpp diffusion level after hub lysosomal inhibition. This new result is now shown in a new Figure 4 as Figures 4K and 4L (shown below) along with characterization of Dpp diffusion.

Moreover, as pointed by this reviewer, we could not detect Dpp mRNA (FISH) as well as Dpp protein (Dpp-mCherry knock-in) in CySCs. We are aware that Jak/STAT signal activation in CySCs has been shown to induce Dpp expression in CySCs (Wang et al., 2008, Leatherman et al., 2010). However, Wang et al., 2008 also did not detect a Dpp signal in situ in wild type testis. Dpp transcription in CySCs was only detected by RT-PCR (Kawase et al), and thus we anticipate that the expression level is too low to detect using an in situ assay.

We added a discussion about this discrepancy in the text as follows;

“It has been reported that dpp mRNA is present in CySC populations [7] and JAK-STAT signaling from hub cells induces an increase in dpp mRNA in CySCs [5, 21]. We tested whether CySC numbers were increased and/or whether CySCs might express a higher amount of dpp mRNA upon lysosome inactivation, which could increase pMad levels in GSCs. We found that the number of CySCs remained the same upon hub specific knock-down of lysosomal genes (Figure S3G-J). A FISH experiment using a fluorescent probe for dpp mRNA showed no change in dpp mRNA levels in the hub upon inactivation of hub lysosomes (Figure S4). Furthermore, we could not detect dpp mRNA in CySCs in either controls or after hub specific knock-down of lysosomal genes (Figure S4), indicating that dpp transcripts are also unlikely to be increased in CySCs. Together, these results suggest that the increased Dpp signal observed after inhibition of hub lysosomes is not caused by increased dpp mRNA levels. Note that the dpp mRNA was also reported to be undetectable in wild type CySCs using in situ method [21]. We speculate that the level of dpp transcript in CySCs must be low such that it is only detectable using reverse transcription (RT)-PCR, as described in the original report [7].”

ii) Gbb levels

It has been reported that Gbb overexpression does not cause any observed defects in testis. Therefore, we focused only on Dpp. We provided a note in the manuscript which reads as follows;

“It should be noted that another BMP ligand, Glass bottom boat (Gbb) has also been reported to bind to Tkv and cooperatively maintain GSCs. Gbb is broadly expressed in somatic cells outside of the niche, whereas Dpp is concentrated in the hub [7]. Unlike ectopic Dpp expression (nosGal4>dpp), which is known to cause germline tumors, Gbb expression (nosGal4>gbb) does not cause any phenotype in the testis [4]. Therefore, we focused on Dpp as a potential rate-limiting factor for the niche-derived BMP signaling in GSCs.”

iii) Involvement of JAK-STAT signal

From new Figure S3E,F and Figure S2E-G.

As pointed out by this reviewer, we realized that increased expression of STAT92E in the germline after failed differentiation has never been documented elsewhere. We examined the amount of STAT92E in a germline tumor expressing a constitutively active form of Tkv (nosGal4>TkvCA). Similar to Spin RNAi and IFT-KD/Tkv-OE testis, we observed an intermediate level of cytoplasmic STAT92E in germline tumor cells (New Figure S2G, right).

This result indicates that enhanced Dpp signaling in the germline leads to high cytoplasmic STAT92E levels (but not nuclear STAT92E levels). We revised the description as follows;

“It should be noted that the cytoplasmic STAT92E level in differentiating germ cells was higher after hub-specific knockdown of lysosomal genes than that of control testes (Figure S3F, G), and similar to testes expressing Tkv-OE/IFT-KD (Figure S2F) or constitutively active Tkv (nosGal4>tkv-CA, Figure S2G), suggesting that enhanced Dpp signal activation results in high STAT92E expression.”

4) IFT-KO and Tkv- The authors have added, as I requested, data showing that the IFT-KD that reduces MT-nanotube length also reduces lysosomal UAS-Tkv in the hub. To answer the other reviewers' concerns about artifacts due to Tkv overexpression, it would have been helpful here to look at effects using the Tkv-GFP-trap instead of UAS-Tkv, as the former is presumably expressed at more endogenous levels. I'd also be curious to see whether or not the authors observed increased Tkv-GFP-trap on the surface of the GSCs after IFT-KD, as this would provide some evidence for or against their new model for the BMP signaling effects in a situation with endogenous Tkv levels.

We could not detect the signal of Tkv-GFPtrap in GSC’s cell body either with or without IFT-KD (Provided the images in new Figure S2A, B). This is likely because of the low level of endogenous Tkv transcription in GSCs. Consistent with this result, we do not see enhanced pMad level (and rather see decreased pMad level) when MT-nanotubes are solely perturbed. We provided the hypothetical model showing how MT-nanotubes can enhance the signal using only small amount of Tkv protein in Figure2L (Figure3L in previous version).

I have one last concern with this section, and my apologies for not asking this in my previous review. The assumption is that IFT-KD is affecting Tkv in GSCs only through its effects on MT-nanotube length, rather than some other mechanism. However, in both ref 6, and Figs 3H and K, UAS-Tkv is strongly increased after IFT-KD in cells quite distant from the hub, and presumably out of range of MT-nanotube-mediated transfer to hub cells. At the best, this suggests a very long-lasting effect on Tkv as cells move away from the hub. At the worst, this suggests there is a completely different mechanism for Tkv stabilization that does not rely on MT-nanotubes. Is there a way to drive IFT-KD in cells only after they lose hub contact, and make sure that Tkv is not affected? If not, then I think some discussion is in order.

We provide the image of BamGal4 driven Tkv-GFP with or without IFT-KD (new Figure S2H, I). IFT-KD did not increase Tkv level in bam positive spermatogonia, suggesting that the IFT-KD-mediated Tkv degradation defect is specifically occurring in GSCs.

Typos:
The Introduction discusses ref 6, but the ref number does not appear until the Results.
"we dpp mRNA"
"pMad leves"
Fig. S1 "Dpp-mChery"

Thank you for pointing out these typos. We have corrected these.

Rev. 2:
The authors did an incredibly thorough job handling every reviewer concern. In particular, I appreciated the live imaging data that was added to clarify the location/cell type of internalized dpp.
